# ART manipulation after controlled ovarian stimulation may not increase the risk of abnormal expression and DNA methylation at some CpG sites of H19,IGF2 and SNRPN in foetuses: a pilot study

**DOI:** 10.1186/s12958-018-0344-z

**Published:** 2018-07-05

**Authors:** Menglu Ji, Xingling Wang, Wenbin Wu, Yichun Guan, Jing Liu, Jingyan Wang, Wenxia Liu, Chunyan Shen

**Affiliations:** Department of Reproductive Medical Center, Third Affiliated Hospital of Zhengzhou University, 7 Kangfuqian Road, Zhengzhou, 450052 Henan People’s Republic of China

**Keywords:** Assisted reproductive technology (ART), Imprinted gene, DNA methylation, Multifoetal reduction, Human foetuses

## Abstract

**Background:**

To examine the effects of IVF, ICSI and FET, as well as in vitro culture, on the safety of offspring, this study was conducted from the perspective of genetic imprinting to investigate whether assisted reproductive technology would influence the parental and maternal imprinting genes.

**Methods:**

Eighteen foetuses were collected from multifoetal reduction and divided into 6 groups: multifoetal reduction after IVF fresh transferred D3 embryos (*n* = 3), multifoetal reduction after IVF frozen transferred D3 embryos (*n* = 3), multifoetal reduction after IVF frozen transferred D5 embryos (*n* = 3), multifoetal reduction after ICSI fresh transferred D3 embryos (*n* = 3), multifoetal reduction after ICSI frozen transferred D3 embryos (*n* = 3), and multifoetal reduction after controlled ovarian hyperstimulation (COH) (*n* = 3). The imprinted genes H19, IGF2 and SNRPN were selected for analysis. The expression and DNA methylation at some CpG sites of H19, IGF2, and SNRPN were examined using real-time quantitative polymerase chain reaction (PCR) and pyrosequencing.

**Results:**

There were no significant differences in the mRNA expression levels among the groups. The mean percentage of H19 methylation (eight CpG sites), IGF2 methylation (five CpG sites) and SNRPN methylation (nine CpG sites) did not differ significantly.

**Conclusions:**

The results suggest that ARTs after controlled ovarian stimulation (IVF, ICSI, cryopreservation and duration of in vitro culture) may not increase the risk of abnormal expression and DNA methylation at some CpG sites of H19, IGF2 and SNRPN in foetuses. Further study with strict design, expanded sample size and CpG sites is essential.

**Electronic supplementary material:**

The online version of this article (10.1186/s12958-018-0344-z) contains supplementary material, which is available to authorized users.

## Background

Since the birth of the first IVF infant in the world in 1978 and the first intracytoplasmic sperm injection (ICSI) infant in 1992, assisted reproductive technology (ART) has grown rapidly, and increasing numbers of babies are born using it. ART has been shown to be associated with preterm birth, low birth weight, congenital malformations and rare imprinting disorders [1]. Therefore, increasing numbers of scholars have begun to pay attention to the safety of ART and have conducted relevant research.

Epigenetic modification is a ubiquitous method of gene regulation in life, and it is crucial to maintaining the normal life activities of mammals. Abnormal epigenetic modification of the embryonic stage may induce a variety of diseases in the embryo or even later in adulthood. DNA methylation is one of the main methods of epigenetic modification. The main modification sites occur on the CpG islands of DNA, which are also susceptible to many environmental factors. In mammals, there are special types of genes called imprinted genes. They are located in differentially methylated regions (DMRs), a particular region of DNA where the maternal and paternal alleles are methylated differentially for the purpose of controlling gene expression [2]. Aberrant expression of imprinted genes triggers a variety of human diseases, affects the growth and development of embryos and foetuses, and induces cancer [3]. During mammalian development, there are two large-scale recoding stages of genomic information, which are primarily changes in epigenetic modifications. The first period is in the primordial germ cell development, when DNA demethylation abrogates epigenetic modifications that are then remethylated during gametogenesis with the addition of the mark. The second period occurs in the preimplantation stage; although the DNA undergoes large-scale demethylation changes during this period, the level of methylation of the imprinted genes does not change. However, ART occurs at the time of the removal and at the establishment and maintenance of the imprinted genes. Therefore, any ART may interfere with the expression of the imprinted genes and may have an impact on the offspring.

Chen et al. [4] found that the in vitro environment may affect the maintenance of gene imprinting, and the H19 gene in human day 3 embryos with poor quality (unsuitable for transplantation or cryopreservation) showed demethylation or hypomethylation patterns after in vitro culture. Wang et al. [5] have reported that mouse embryo vitrification tests exacerbate the H19 gene loss and increase expression. A recent meta-analysis found that any imprinting disorder such as Angelman syndrome, Prader-Willi syndrome and Beckwith-Wiedemann syndrome in children conceived through ART had almost fourfold higher incidence than that in children conceived naturally [6]. While overcoming reproductive disorders, ART has the potential to affect the methylation process and affect the health of offspring [7].

Due to the scarcity of embryos used for research and the related ethical restrictions, few reports have been reported on the gene expression of early human foetuses. Most studies use animal models to assess early embryonic development. To the best of the authors’ knowledge, no study so far has addressed the impact of various stages of ART on imprinted genes. Therefore, 18 foetuses from multifoetal reduction were collected and divided into 6 groups. H19, IGF2 and SNRPN, which are well-studied imprinted genes, were selected for analysis. In humans, H19 is paternally imprinted, whereas IGF2 and SNRPN are maternally imprinted. The expression and DNA methylation of genes were analysed using real-time quantitative polymerase chain reaction (PCR) and pyrosequencing. The aim of the present study was to discuss the impact of ART on the safety of offspring from the perspective of genetic imprinting.

## Methods

### Sample collection

The study protocol was approved by the Third Affiliated Hospital of Zhengzhou University Institutional Ethics Committee, and informed consent was obtained from all patients. Between January 2014 and December 2016, 18 multifoetal reduction foetuses were obtained from 18 patients respectively for this study. The inclusion criteria for this study were single gestational sacs and 6–9 weeks of pregnancy. The preoperative B ultrasounds showed intrauterine live births. Preoperative examinations of all the patients were carried out with routine blood examinations, routine urine analysis, routine leukorrheal analysis, hepatic and renal functions tests, infectious diseases examinations, TORCH screens, coagulation analyses and other tests. All the results were normal. During the reduction, the principle of aseptic technique was strictly observed, and the foetal fraction was aspirated first if possible. Then, further confirmation was obtained using the OLYMPUS SZX16 microscope to remove other parts such as the chorionic tissue. Finally, the foetuses (the foetal fraction) were rinsed under iced saline to wash off the blood and then packed for cryopreservation in liquid nitrogen. The foetuses were transferred to a − 80 degree C refrigerator for later use (AllProtect™ Nucleic Acid and Protein Stabilization Reagent for Animal Tissue was added).

All the samples were divided into six groups according to the source as follows: 1) multifoetal reduction after IVF fresh transferred D3 embryos (*n* = 3); 2) multifoetal reduction after IVF frozen transferred D3 embryos (*n* = 3); 3) multifoetal reduction after IVF frozen transferred D5 embryos (*n* = 3); 4) multifoetal reduction after ICSI fresh transferred D3 embryos (*n* = 3); 5) multifoetal reduction after ICSI frozen transferred D3 embryos (*n* = 3); and 6) multifoetal reduction after controlled ovarian stimulation (COS) (*n* = 3). Patients in groups 1–5 were those who received assisted reproductive therapy in the study centre; patients in group 6 were those who had ovulatory disorders and were treated with controlled ovarian stimulation in primary hospitals followed by spontaneous pregnancy, and all patients performed multifoetal pregnancy reduction in the study centre. The patient characteristics are shown in Table 1.

**Table 1:** Characteristics of multifoetal reduction patients

Sample no.	Source	Maternal age(y)	Gestational age(d)
1	IVF-ET-D3	35	52
2	IVF-ET-D3	32	51
3	IVF-ET-D3	28	53
4	IVF-FET-D3	28	49
5	IVF-FET-D3	36	48
6	IVF-FET-D3	28	50
7	IVF-FET-D5	30	50
8	IVF-FET-D5	30	50
9	IVF-FET-D5	29	52
10	ICSI-ET-D3	32	56
11	ICSI-ET-D3	27	54
12	ICSI-ET-D3	25	55
13	ICSI-FET-D3	26	52
14	ICSI-FET-D3	33	59
15	ICSI-FET-D3	35	51
16	COS	31	50
17	COS	28	55
18	COS	29	59

Comparisons between groups 1–5 and group 6 were mainly used to investigate the effects of ART on foetuses after controlled ovarian stimulation; comparisons between group 1 and group 2 and between group 4 and group 5 were used to research the effect of cryopreservation on foetuses; comparisons between group 1 and group 4 and between group 2 and group 5 were used to study the effect of IVF and ICSI on the foetuses; and the comparison between group 2 and group 3 was used to examine the effect of the duration of in vitro culture.

### RNA expression analysis

Using the mirVana miRNA Isolation Kit (Ambion), total RNA was isolated from the foetus according to the manufacturer’s instructions and checked with an Agilent 2100 Bioanalyzer. Then, the ReverTra Ace qPCR Kit (TOYOBO) was used for reverse transcription as described by the manufacturer (ref). The reverse transcription reaction started with 15-min at 37 °C, ended after a 5-min at 98 °C and was held at 4 °C. PCR was performed with one denature cycle at 50 °C for 2 min and 95 °C for 10 min, 40 amplification cycles at 95 °C for 15 s and 60 °C for 1 min, using ABI Power SYBR Green PCR Master Mix (ABI, USA) on the 7900 HT Sequence Detection System (ABI, USA) according to the manufacturer’s protocol. The housekeeping gene Gapdh was used as the reference gene. Finally, the dissolution curve was added, and the result of the dissolution curve analysis showed a single peak, indicating that the PCR amplification specificity was excellent. The repeatability of the data of three replicates was good. Data were analysed using the ΔΔCt method. Primer sequences for these genes are shown in Table 2.

**Table 2:** Primers for real-time quantitative polymerase chain reaction (PCR)

Gene		Primer sequence(5’to3’)		Product size(bp)
H19	ID:283120	Forward	TCAAAGCCTCCACGACTCTGT	88
		Reverse	GCCGTCTCCACAACTCCAA	
IGF2	ID:3481	Forward	TGAGATCCAAAACGCTTCGA	87
		Reverse	CGGCGAGGCAGAATATAACAC	
SNRPN	ID:6638	Forward	CGTCCACCAAGACCTTAGCATAC	100
		Reverse	AACAAAAAGCTCACAAACACTCTACAC	
Gapdh	ID:2597	Forward	TGACTTCAACAGCGACACCCA	100
		Reverse	CACCCTGTTGCTGTAGCCAAA	

### DNA methylation analysis

The promoter region sequences of targeted imprinted genes H19, IGF2 and SNRPN were searched from UCSC and NCBI and imported into PyroMark Assay Design 2.0 software for primer design. According to the primer design scores, Tm and %GC, the corresponding primer sequences were designed for the three imprinted genes. Primer sequences used for pyrosequencing and the analysed sequences are shown in Additional file 1.

Genomic DNA was extracted using the OMEGA TISSUE DNA Kit (200) according to the manufacturer’s instructions and quantified using agarose gel electrophoresis before the follow-up reaction. Each 0.5–1 μg DNA sample was transformed with the EZ DNA Methylation-Gold ™ Kit. Using the transformed DNA sample as a template, PCR amplification was performed as described by manufacturer (ref). Thermocycling conditions consisted of 35 cycles of 98 °C for 10 s, 55 °C for 30 s, and 72 °C for 30 s; then a one-minute extension at 72 °C, and finally samples were held at 4 °C. Pyrosequencing was performed on the PyroMark Q96 Real-Time Quantitative Pyrosequencing Analyser (Qiagen) by taking 15–20 μL of PCR products for each sample.

### Statistical analysis

All statistics were performed with IBM SPSS Statistics 23.0. Quantitative data were calculated as the mean ± SD. The Kruskal-Wallis H test was used to evaluate the results among the six groups. A difference was considered significant when *P* ≤ 0.05.

## Results

### Patient characteristics

A total of 18 foetuses from multifoetal reduction of 18 patients respectively were included in this study. The specific grouping was as described above. There was no significant difference in the maternal age between all groups (*P* = 0.791); and there was also no significant difference in gestational age between groups (*P* = 0.068).

### Changes in the expression levels of genes

The result showed that no significant differences in expression were detected for H19, IGF2 and SNRPN genes (H19: *P* = 0.688; IGF2: *P* = 0.527; SNRPN: *P* = 0.295). The results for gene expression are shown in Table 3.

**Table 3:** The 2^-ΔCt^ value of genes among groups

Value of 2^-ΔCt^	Group 1 (*n* = 3)	Group 2 (*n* = 3)	Group 3 (*n* = 3)	Group 4 (*n* = 3)	Group 5 (*n* = 3)	Group 6 (*n* = 3)
H19	1.06 ± 0.15	1.31 ± 0.20	1.62 ± 0.94	1.22 ± 0.58	1.29 ± 0.61	1.36 ± 0.07
IGF2	0.59 ± 0.01	0.46 ± 0.14	0.81 ± 0.56	0.58 ± 0.18	0.47 ± 0.23	0.65 ± 0.14
SNRPN	0.13 ± 0.01	0.11 ± 0.03	0.16 ± 0.03	0.14 ± 0.01	0.14 ± 0.04	0.12 ± 0.02

Group 1: multifoetal reduction after IVF fresh transferred D3 embryos (*n* = 3); Group 2: multifoetal reduction after IVF frozen transferred D3 embryos (*n* = 3); Group 3: multifoetal reduction after IVF frozen transferred D5 embryos (*n* = 3); Group 4: multifoetal reduction after ICSI fresh transferred D3 embryos (*n* = 3); Group 5: multifoetal reduction after ICSI frozen transferred D3 embryos (*n* = 3); Group 6: multifoetal reduction after controlled ovarian stimulation (COS) (*n* = 3)

### DNA methylation status of H19, IGF2 and SNRPN

The mean percentage of H19 methylation (eight CpG sites), IGF2 methylation (five CpG sites) and SNRPN methylation (nine CpG sites) showed no significant differences in all groups (H19: *P* = 0.169; IGF2: *P* = 0.058; SNRPN: *P* = 0.748). The specific mean percentages of gene methylation is shown in Table 4.

**Table 4:** The mean percentage of gene methylation among groups

Mean value of methylation (%)	Group 1 (*n* = 3)	Group 2 (*n* = 3)	Group 3 (*n* = 3)	Group 4 (*n* = 3)	Group 5 (*n* = 3)	Group 6 (*n* = 3)
H19	38.21 ± 7.10	36.58 ± 6.66	37.79 ± 8.84	38.96 ± 7.37	32.67 ± 11.91	39.21 ± 7.10
IGF2	1.87 ± 1.25	3.73 ± 2.22	3.73 ± 2.96	2.33 ± 1.29	3.07 ± 3.63	1.93 ± 0.59
SNRPN	45.96 ± 5.47	47.67 ± 2.77	47.19 ± 3.01	46.93 ± 3.52	46.63 ± 3.83	47.44 ± 3.50

Group 1: multifoetal reduction after IVF fresh transferred D3 embryos (*n* = 3); Group 2: multifoetal reduction after IVF frozen transferred D3 embryos (*n* = 3); Group 3: multifoetal reduction after IVF frozen transferred D5 embryos (*n* = 3); Group 4: multifoetal reduction after ICSI fresh transferred D3 embryos (*n* = 3); Group 5: multifoetal reduction after ICSI frozen transferred D3 embryos (*n* = 3); Group 6: multifoetal reduction after controlled ovarian stimulation (COS) (*n* = 3)

## Discussion

Although ART is an important method in the treatment of many infertile couples, the safety of these procedures has raised many concerns. Hormone stimulation, in vitro fertilization, intracytoplasmic sperm injection, cryopreservation, the timing of embryo transfer and many other techniques in ART must occur in a specific window of time to establish and maintain the correct genomic imprints. Whether these large numbers of non-physiological operations lead to the abnormal epigenetic modification of the embryo and whether it will increase the risk of foetal defects are the important issues that need to be thoroughly investigated.

Some studies indicated an increased risk of birth defects in ART [8, 9], while others showed the opposite [10–12]. It remains a controversial question. The mechanisms that cause imprinted abnormalities in ART offspring are unclear. Possible factors include infertility itself, ovulation induction, mechanical damage of IVF/ICSI, cryopreservation and in vitro culture. It has been shown that superovulation affects the expression of imprinted genes. Loss of SNRPN and gain of H19 imprinted methylation were observed in superovulation [13]. Animal experiments [14] showed that imprinted genes H19 and SNRPN were unaffected in either the placentae or the embryos from the superovulated females. However, the paternally expressed imprinted gene IGF2 had significantly more variable mRNA levels. Due to the application of hormones, superovulation will interfere with the status of imprinted genes on account of the non-physiological endocrine environment. However, the impact of various stages of ART after superovulation on the imprinted genes remains to be investigated, so this study was conducted.

At present, there are not enough reports on human embryo methylation, especially on the methylation status of imprinted genes after embryo implantation of ART. This pilot study collected multifoetal reduction foetuses for examination, thus offsetting the deficiencies in this area. Due to the different time of establishment and different sensitivity to external factors in imprinted genes, the parental imprinting gene H19 and maternal imprinting genes IGF2 and SNRPN were selected to conduct a more comprehensive analysis on the impact of different steps of ART on the foetus.

In this study, there were no significant differences in the maternal age and gestational age, indicating that the collected samples were well balanced. None of the 3 studied imprinted genes (H19, IGF2 and SNRPN) differed significantly in the mRNA expression levels and the mean percentage of methylation at some CpG sites. It illustrated that cryopreservation may have no negative effect on the foetus (group 1 vs. group 2; group 4 vs. group 5). Similar to the current findings, Derakhshan-Horeh et al. reported that the H19/IGF2 DMR methylation status of day 3 embryos appeared not to be affected by vitrification [15]. As for the duration of in vitro culture, one study that considered extended culture did not show that it posed a greater risk for imprinting errors than short culture [16]. The same conclusion was reached when comparing group 2 with group 3. Mechanical manipulations of IVF and ICSI also did not result in changes in imprinted genes (group 1 vs. group 4; group 2 vs. group 5). In addition, the comparisons between groups 1–5 and group 6 further demonstrate that post-superovulation manipulations have no effect on DNA methylation of the imprinted gene and do not cause a change in the corresponding mRNA levels. The IGF2 gene (five CpG sites) exhibited a slight difference in the mean percentage of methylation among groups (*P* = 0.058), but it was not statistically significant. Owing to the fact that imprinted genes can show considerable methylation variation among normal individuals [17], it is difficult to judge whether this phenomenon is caused by individual differences or caused by ART technology. The sample size must be further expanded to conduct more effective research.

Previous studies have demonstrated that children conceived by ART do not show a higher degree of imprint variability and do not have a higher risk of DNA-methylation defects [11, 18, 19], which were consistent with the results of the current study. A study conducted by Li et al. found no significant increase in imprint variability at H19/IGF2 DMR [20]. Another study from Wong et al. showed that ART might not affect proper imprinting of H19 and IGF2 in the placenta [21]. However, Sakian et al. reported that H19 expression was significantly increased in both IVF and ICSI placentas and IGF2 was significantly decreased when compared to controls [22]. However, these studies did not discuss the role of superovulation and the mechanical manipulation of IVF and ICSI separately, so it is not possible to differentiate whether hormone-induced or ART technology itself made changes in gene expression. This research takes this point into full consideration.

In the past, a large number of studies used the BSP sequencing method. Although this method can clearly show the methylation status of each CpG site in the promoter region, it requires large amounts cloning, complicated operations, it is expensive and it is difficult to carry out en masse. In addition, the degree of methylation quantified depends on the number of clones selected, so this method can only be regarded as a semi-quantitative technology. Pyrosequencing, however, is characterized by its high throughput, low cost, rapidity and visualization. Pyrosequencing is a sequencing-by-synthesis method that allows the accurate evaluation of DNA methylation at CpG sites with high quantitative resolution [23]. However, the length of the sequenced fragment is relatively short. The current study used this new method to study the methylation status of imprinted genes. Although fewer CpG sites were selected, their methylation status could be reflected in a more realistic and accurate way. Further research is needed to expand the studied scope of CpG sites in imprinted gene promoter regions.

This research shows that ARTs after controlled ovarian stimulation (IVF, ICSI, cryopreservation and duration of in vitro culture) may not increase the risk of imprinting defects in the offspring. The increase in imprinted diseases does not seem to be directly affected by ART but the increased fertility problems of the parents [24] or the use of superovulation hormone. This hypothesis should be confirmed by further study. Another possible reason for these research results is that the use of good-quality embryos for transplantation was not excluded. Poor-quality embryos with a high methylation error rate [25] have been eliminated artificially by the time of initial transplantation.

Although the current study suffers from a small sample size (3 foetuses in each group), it is the first research to discuss the post-superovulation procedures in ART separately. Further studies are needed to extend the analysis to more foetuses and subjoin the quality of the transplanted embryos.

## Conclusions

To date, ART has been born and has been used clinically for 39 years. The impact of ART on offspring has not yet been clearly revealed. For infertile couples, the most important thing is to nurture a healthy baby. This article only discusses the effect of ART on DNA methylation, and no significant abnormality was found at some CpG sites. Although the results are reassuring, the limitations of this study suggest that additional large studies are needed. Furthermore, other epigenetic mechanisms such as histone post-translational modifications and chromatin remodelling have been implicated in the regulation of these imprinted genes [26, 27]. These also remain to be elucidated. Thus, the safety of ART should be investigated in further studies, and a stricter study design is essential.

## Additional file

Additional file 1: Sequences of primers used for pyrosequencing reactions and the sequence to analyse. (DOCX 18 kb)

## Abbreviations

ART: Assisted reproductive technology
FET: Frozen embryo transfer
ICSI: Intracytoplasmic sperm injection
IVF: In vitro fertilization
PCR: Polymerase chain reaction
