# Supplementary material for: ART manipulation after controlled ovarian stimulation may not increase the risk of abnormal expression and DNA methylation at some CpG sites of H19,IGF2 and SNRPN in foetuses: a pilot study
Source: Reprod Biol Endocrinol. 2018 Jul 5;16:63. doi: 10.1186/s12958-018-0344-z (PMC6034287; doi:10.1186/s12958-018-0344-z)
Supplement: Supplementary file 1 — Sequences of primers used for pyrosequencing reactions and the sequence to analyse. (DOCX 18 kb) [file 12958_2018_344_MOESM1_ESM.docx]

| Additional file 1 | | | | |
| --- | --- | --- | --- | --- |
| Sequences of primers used for pyrosequencing reactions and the sequence to analyze. | | | | |
| Gene | **Primer** | **Sequence(5'to3')** | **Sequence to analyze(5'to3')** | **Number of CpGs** |
| H19 | Forward | GGGTTTGGGAGAGTTTGTG | TYGTTTATYG TTTGTTAGTA GAGTGYGTTY GYGAGTYGTA AGTATAGTTY GGTAATATGY GGTTTTTAGA TAGGAA | 8 |
|  | Reverse* | TTCCCCAAAACAAAATCCCCACAAC |  |  |
|  | Sequencing | GGAGAGTTTGTGAGG |  |  |
| IGF2 | Forward | GTTTGAGGTTAAGAAGGGTAGAGT | TTYGATTYGG AGAGAGGTYG YGGTTTTTGT TTAGTGGGTA GYGTGGAAGT TTTTATATAA GGAGGTGG | 5 |
|  | Reverse* | AAAAAAATCTCCTTCCCACCTCCTTATAT |  |  |
|  | Sequencing | GTTAAGAAGGGTAGAGTT |  |  |
| SNRPN | Forward | GAGGGAGTTGGGATTTTTGT | AYGTTTGYGY GGTYGTAGAG GTAGGTTGGY GYGTATGTTT AGGYGGGGAT GTGTGYGAAG TTTGTYGTTG TTGTAG | 9 |
|  | Reverse* | CCACCCACACAACTAACCTTACCC |  |  |
|  | Sequencing | TTTTGTATTGAGGTAAATAAGT |  |  |
| Biotinylated primers are indicated by an asterisk.  “Y” represents the CpG site. | | | | |
